# Implementation of Open PCR System for the Detection of TB/DR-TB and NTM in Sputum Samples from Suspected Pulmonary Tuberculosis Patients in Medan, Indonesia

**DOI:** 10.3390/tropicalmed11060168

**Published:** 2026-06-18

**Authors:** R Lia Kusumawati, Mirzan Hasibuan, Nisrina Tari, Gema Nazri Yanni, Laura Isa Ginting, Cynthia Gozali, Tryna Tania

**Affiliations:** 1 Department of Microbiology, Faculty of Medicine, Universitas Sumatera Utara, Medan 20155, Indonesia; 2 Prof. Dr. Chairuddin Panusunan Lubis Universitas Sumatera Utara Teaching Hospital, Universitas Sumatera Utara, Medan 20155, Indonesia; mirzanhasibuan@usu.ac.id (M.H.); gema.nazri.yanni@usu.ac.id (G.N.Y.); lauraisaginting97@gmail.com (L.I.G.); 3 PCR-WGS Microbiology Laboratory, Faculty of Medicine, Universitas Sumatera Utara, Medan 20155, Indonesia; nisrinatari21@gmail.com; 4 Department of Pediatrics, Faculty of Medicine, Universitas Sumatera Utara, Medan 20155, Indonesia; 5 Division of Materials Science, Graduate School of Science and Technology, Nara Institute of Science and Technology, 8916-5 Takayama, Ikoma, Nara 630-0192, Japan; cynthiagozali8@gmail.com; 6 Clinical Microbiology, RS Cipto Mangunkusumo, Jakarta 10430, Indonesia; tryna.tania@gmail.com

**Keywords:** tuberculosis, *Mycobacterium tuberculosis*, nontuberculous mycobacteria, isoniazid resistance, open PCR system, TB-NTM coinfections

## Abstract

(1) Background: Indonesia faces the dual challenge of a high tuberculosis (TB) burden and increasing drug resistance. Conventional molecular diagnostics frequently fail to detect isoniazid resistance and nontuberculous mycobacteria (NTM). This study evaluates a domestic multiplex Open PCR system in Medan, Indonesia. (2) Methods: From July to November 2025, 1569 sputum specimens from suspected TB patients were analysed using the Indigen MTB/NTM/DR-TB Real-time PCR Kit Gen 2. (3) Results: Mycobacterial DNA was detected in 421 specimens (26.8%). Among these, 396 (94.1%) were drug-susceptible TB, while 16 (3.8%) showed resistance, predominantly INH mono-resistance (*n* = 14; 0.89% of total). Additionally, 9 cases (2.1%) involved NTM or TB-NTM co-infections. Tertiary hospitals showed significantly higher positivity rates (33.5%) than primary care (18.9%; *p* < 0.001). TB status was significantly associated with male (*p* = 0.0052) and older age (*p* = 0.006), whereas resistance profiles and NTM distribution were consistent across all demographic groups (*p* > 0.80). (4) Conclusions: This study describes the implementation and diagnostic yield of a domestic multiplex Open PCR system in Medan, Indonesia. By bridging diagnostic gaps across a decentralized referral network, this facilitates rapid and targeted therapy. Integrating multiplex domestic innovations into national diagnostic algorithms is essential for achieving Indonesia’s TB elimination targets.

## 1. Introduction

Tuberculosis (TB) continues to pose a major global public health challenge and remains the leading cause of death from a single infectious agent. Although international interventions have been implemented, progress toward the 2030 elimination targets has been inadequate in most high-burden regions. In 2024, an estimated 10.7 million individuals fell ill with TB globally, resulting in 1.23 million deaths. Indonesia ranks second globally in TB burden according to the WHO Global Tuberculosis Report 2025 [1,2]. North Sumatra represents a critical TB hotspot, ranking third nationally after West Java and East Java, with an estimated 74,434 cases in 2024 [3]. Within the province, Medan reports the highest number of cases among regencies and cities, with 10,050 cases in 2022, likely influenced by its high population density [4,5].

Indonesia’s TB diagnostic framework relies heavily on the Xpert MTB/RIF assay, but it is limited to detecting only rifampicin (RIF) resistance [6,7]. In contrast, the emergence of multidrug-resistant TB (MDR-TB) necessitates broader surveillance, particularly for Isoniazid (INH) resistance and the presence of Nontuberculous Mycobacteria (NTM), which are frequently misidentified as TB in clinical settings [8,9]. The Indigen MTB/NTM/DR-TB Real-time PCR Kit Gen 2 addresses these gaps by detecting not only MTB and NTM but also drug resistance markers for RIF (RIF A, RIF B) and INH (inhA, katG), offering a more comprehensive diagnostic profile than closed systems [10].

In response to the 2022 TB Joint External Monitoring Mission (JEMM) recommendations [11], Indonesia’s National Tuberculosis Control Program prioritized diagnostic diversification by utilizing the post-COVID molecular testing network. Approximately 1046 laboratories nationwide possess real-time Open PCR capacity, providing a rapid, scalable, and cost-effective platform for TB diagnosis. This transition is supported by Presidential Decree No. 67/2021 and Presidential Instruction No. 2/2022, which both emphasize intensified TB case finding and the adoption of locally made components (TKDN). Building on these policy directives, the Ministry of Health initiated operational research in 23 districts across 8 provinces to evaluate domestic multiplex PCR kit, including Indigen MTB/NTM/DR-TB Gen 2, for TB detection in sputum-suitable suspected cases [12].

Integrated into this national framework, the Prof. Dr. Chairuddin P. Lubis Universitas Sumatera Utara (USU) Hospital was officially designated by the Ministry of Health in July 2025 as a reference hospital for Open PCR TB diagnostics following completion of the operational research. Since then, the facility has provided free Open PCR TBC diagnostic services to the public, significantly strengthening the detection capacity in Medan. By integrating advanced multiplex molecular assays, the site plays a critical role in mitigating the nation’s diagnostic gap, providing the evidence base necessary for targeted clinical interventions in high-burden settings while simultaneously advancing the molecular research necessary to combat drug-resistant TB.

## 2. Materials and Methods

### 2.1. Study Design, Clinical Specimen Collection, and Ethical Considerations

This study was conducted as part of a national initiative to implement Open PCR systems for TB diagnostics. In accordance with the national spot-morning or spot-spot protocol, two specimens were obtained from each patient: the first spot specimen at the initial clinical visit and the second spot-morning specimen collected immediately upon waking the following day, before oral hygiene or food intake [12]. A volume of 1 mL mucopurulent sputum per container was required to ensure sufficient material for molecular analysis. Ethical clearance was obtained from the Health Research Ethics Committee of Universitas Sumatera Utara] (No: 397/KEPK/USU2025).

### 2.2. Molecular Detection via Indigen MTB/NTM/DR-TB Assay

Molecular assay was performed using the Indigen MTB/NTM/DR-TB Real-time PCR Kit Gen 2 (PT Kalgen DNA, Jakarta, Indonesia) [10], designed to detect *Mycobacterium tuberculosis* (MTB), Nontuberculous Mycobacteria (NTM), and mutations in four gene regions associated with resistance to Rifampicin (RIF A, RIF B) and Isoniazid (inhA, katG).

#### 2.2.1. DNA Extraction

DNA was isolated using a chemical liquefaction and thermal lysis protocol. Sputum samples (750 μL) were liquefied with an equal volume of sputum liquefaction solution and incubated for 30 min at room temperature with periodic homogenization. Following centrifugation at 14,000 rpm, the resulting pellet was washed with TB Washing Solution. For lysis, the pellet was resuspended in 200 μL of TB Solution A and supplemented with 2 μL of TB Internal Control. Thermal lysis was executed at 100 °C for 30 min using a secured heat block. After a final centrifugation (10 min at 14,000 rpm), the DNA-containing supernatant was obtained and stored at −20 °C until PCR amplification process.

#### 2.2.2. Real-Time PCR Amplification

Two master mixes for PCR Mix A and PCR Mix B, were prepared for each sample to accommodate the multiplex nature of the assay. Each 20 μL reaction volume consisted of 18 μL of Master Mix (comprising TB Reagent A/B, Enzyme Mix, and NFW) and 2 μL of DNA template. Standardized Positive and Negative Controls were included in every run. The reactions were processed using the Rotor-Gene Q (Qiagen, Hilden, Germany) real-time platform.

### 2.3. Data Interpretation

Data acquisition and analysis were performed using the Rotor-Gene Q software (v2.3.1). Cycle threshold (Ct) values were analyzed across four fluorescent channels. Results were interpreted based on the exponential amplification phase of the fluorescence curves, with visual verification of the auto-threshold settings. The presence of MTB, NTM, and specific drug-resistance alleles was determined by comparing Ct values against the manufacturer’s standardized thresholds for each target.

### 2.4. Data Analysis

Descriptive statistics were used to characterize the study population and diagnostic yield. Associations between demographic variables and PCR outcomes were analyzed using R Studio (v4.6.0), with statistical significance set at *p* < 0.05.

## 3. Results

### 3.1. Study Population and Patient Demographic Distribution

**Figure 1:**
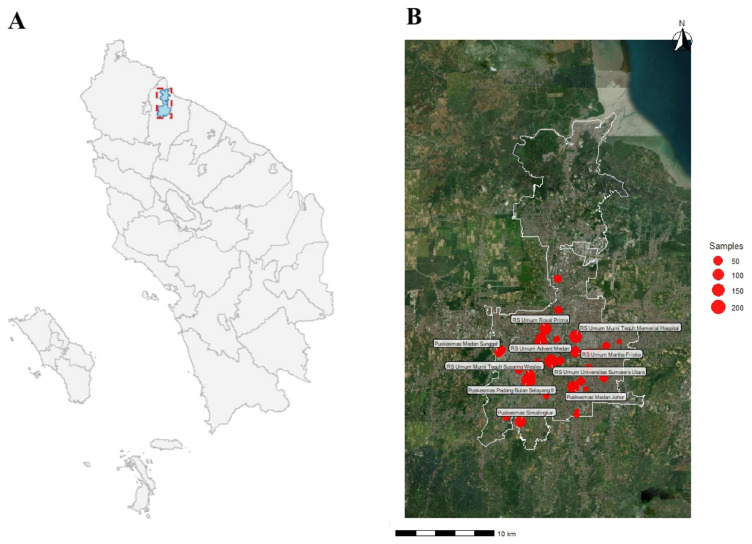
Geographic distribution of the 35 participating healthcare facilities in Medan, North Sumatra. Panel (**A**) shows the administrative map of Medan, while Panel (**B**) shows the spatial distribution of participating facilities and their relative specimen contributions during the study period.

During the study period, a total of 1569 presumptive TB patients were enrolled and screened using the Open PCR system at the Prof. Dr. Chairuddin P. Lubis USU Hospital. This study population was derived from a broad network of 35 participating healthcare facilities distributed across Medan City (Figure 1). As illustrated in Figure 1B, the geographic origin of these referrals spans the entire metropolitan area.

While referrals were widespread, the contribution of specimen volume was heterogeneous among participating sites. High-volume contributors included both primary and tertiary facilities, notably Puskesmas Padang Bulan Selayang II (*n* = 214), the reference Prof. dr. Chairuddin P. Lubis USU Hospital (*n* = 180), and Murni Teguh Memorial Hospital (*n* = 144). Site-specific data available in Supplementary Data S1.

The study population was predominantly male (*n* = 899; 57.3%) compared to female participants (*n* = 670; 42.7%). The mean age was 47.6 ± 19.0 years, reflecting a wide demographic range within the referred cohort (Table 1).

**Figure 2:**
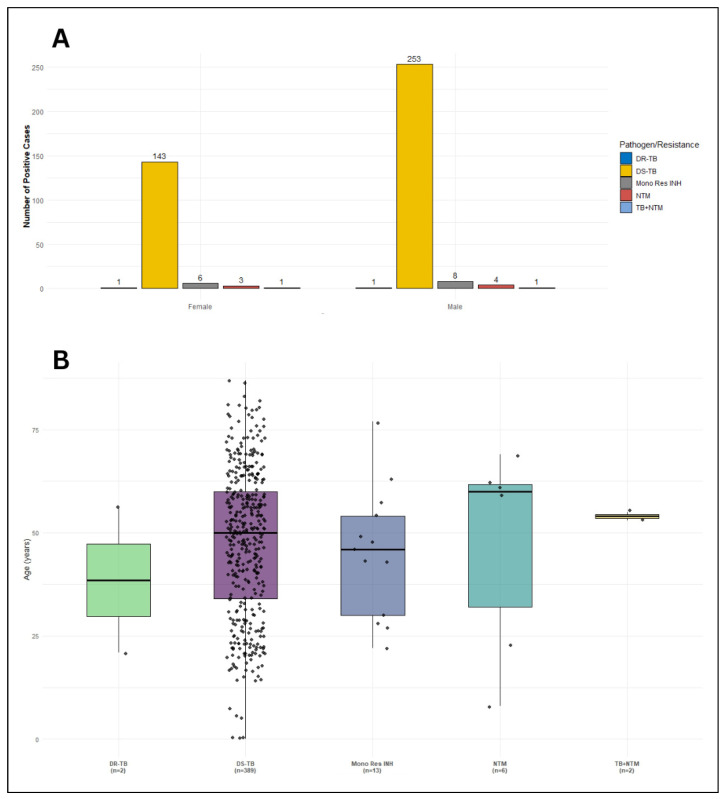
Diagnostic profiles and demographic distribution of mycobacterial-positive cases (*n* = 421). (**A**) Distribution of Open PCR diagnostic categories by sex. (**B**) Age distribution across diagnostic categories shown using scatter and boxplots. Dots represent individual observations, boxplots indicate the median and interquartile range (IQR), and whiskers indicate the spread of the data. Abbreviations: DS-TB, drug-susceptible tuberculosis; DR-TB, drug resistance-associated tuberculosis profile detected by the assay; Mono-Res INH, isoniazid resistance-associated targets only; NTM, nontuberculous mycobacteria; TB + NTM, co-detection of MTB and NTM signals.

Statistical analysis of positive cases (*n* = 421) identified a significant difference in age distribution by sex, as shown in Figure 2. Male patients had a significantly higher median age than female patients (51 years versus 46 years; Wilcoxon rank-sum test, *p* = 0.006). In contrast, no significant age differences were identified across diagnostic categories (Kruskal–Wallis test, *p* = 0.867). Furthermore, sex was not significantly associated with the specific PCR result (Fisher’s exact test, *p* = 0.891). These results indicate that although age profiles differ by sex within the positive cohort, the type of infection, whether DS-TB, DR-TB, or NTM, is independent of these demographic factors in the Medan patient population.

### 3.2. Open PCR Detection Profile in TB and NTM Screening

Out of the 1569 specimens analyzed, 1148 (73.17%) were negative for mycobacterial DNA, while 421 (26.8%) came out positive for MTB, NTM, or TB-NTM co-infections (Table 2). Among the positive cohort, the vast majority were identified as drug-susceptible TB (DS-TB; *n* = 396; 25.24% of total). A key strength of the Indigen assay was its ability to differentiate drug resistance and NTM species effectively. Drug resistance was identified in 16 patients, dominated by Isoniazid (INH) mono-resistance (*n* = 14; 0.89%), with only two cases (0.13%) classified as multidrug-resistant (DR-TB). Furthermore, the system identified 9 cases involving NTM (7 pure NTM; 2 TB-NTM co-infections). The detection of INH mono-resistance and NTM, which would likely be mismanaged under a Rifampicin-only screening algorithm, underscores the importance of clinical utility of the Open PCR system in this endemic setting.

**Figure 3:**
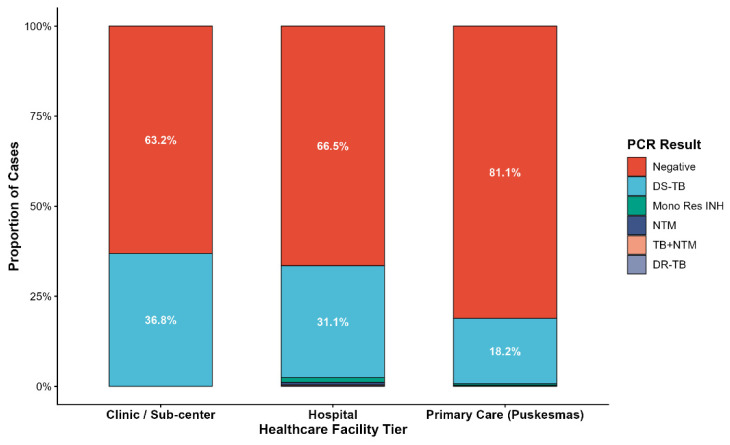
Distribution of Open PCR diagnostic categories by healthcare facility tier. Stacked bars show the proportion of negative results, DS-TB, DR-TB, Mono-Res INH, NTM, and TB + NTM across clinic/sub-center, hospital, and primary care (Puskesmas) facilities. Abbreviations: DS-TB, drug-susceptible tuberculosis; DR-TB, drug resistance-associated tuberculosis profile detected by the assay; Mono-Res INH, isoniazid resistance-associated targets only; NTM, nontuberculous mycobacteria; TB + NTM, co-detection of MTB and NTM signals.

Diagnostic results varied significantly according to healthcare facility type (χ^2^ = 42.871, *p* = 4.904 × 10^−10^), as shown in Figure 3. Tertiary hospital-based facilities reported the highest number of positive detections, with 278 of 830 samples (33.5%) testing positive. This likely reflects a pre-selected patient population with more severe or classic symptoms of pulmonary TB. Primary care facilities (Puskesmas) submitted the largest number of specimens (*n* = 720) but demonstrated a lower positivity rate (136 of 720; 18.9%). This is typical for primary care settings where screening is broader and may include individuals with milder or more non-specific respiratory symptoms. Clinic and sub-center facilities showed 7 positive results among 19 specimens (36.8%). This estimate should be interpreted with caution due to the small sample size hence the percentage is less stable than the other tiers.

The reference hospital served as a pivotal diagnostic hub, contributing the second-largest sample volume and the highest number of DS-TB detections (*n* = 47). There is a marked difference in positivity rates between primary care and tertiary hospitals. For example, Puskesmas Padang Bulan reports a positivity rate of approximately 14%, whereas RS Umum Royal Prima demonstrates a substantially higher rate of 40%. These results reflect the higher clinical suspected cases and disease severity typical of hospital-based referrals. These differences likely reflect referral patterns and case-mix variation across healthcare tiers rather than intrinsic differences in assay behavior. Accordingly, facility-level positivity should not be interpreted as a direct indicator of assay performance.

## 4. Discussion

The Open PCR system demonstrated effective case identification across all healthcare facilities in Medan, with the highest detection rates observed in hospital settings. The lower positivity rate at the Puskesmas level indicates the system’s broad screening capacity within the community, successfully ruling out approximately 81% of suspected cases. Conversely, the concentration of confirmed cases in tertiary settings justifies the Ministry of Health’s strategy to prioritize molecular infrastructure in reference hospitals to manage the complex diagnostic burden of North Sumatra (see Supplementary Data S2 for diagnostic outcomes based on healthcare facility).

A critical finding of this study is the detection of NTM and TB-NTM co-infections. In many TB-endemic regions, NTM infections are frequently misdiagnosed as MDR-TB due to overlapping clinical presentations and the limitations of standard GeneXpert MTB/RIF assays, which only detect MTB [13,14]. Our detection of NTM-related signals highlights the potential presence of NTM among presumptive TB cases; however, molecular detection alone does not establish NTM pulmonary disease. Clinical interpretation requires correlation with symptoms, radiology, and, where possible, repeated microbiological confirmation according to ATS/ERS/ESCMID/IDSA criteria [15,16].

While the WHO-recommended Line Probe Assay (LPA) can differentiate MTB from NTM, its technical complexity and 6 h processing time often delay clinical decisions [17]. The Open PCR platform implemented here provides simultaneous molecular detection of MTB, NTM-related signals, and INH/RIF resistance-associated targets with relatively rapid workflow. However, the assay does not provide species-level NTM identification, which remains necessary for clinical management and epidemiological interpretation. This diagnostic capability facilitates the transition toward NGS-based surveillance to map NTM species-level diversity circulating in Medan. As evidenced by regional findings in Java, where the *M. fortuitum* and *M. abscessus* groups were predominant, precise identification is critical, as these pathogens necessitate therapeutic interventions that deviate significantly from standard anti-tuberculosis regimens [18].

Our analysis revealed a significant association between gender and TB status, with males exhibiting higher positivity rates and being significantly older than females. This male predominance likely reflects cumulative risk factors prevalent in the Indonesian male population, such as high rates of tobacco use and occupational exposure [19,20]. However, once infection was established, the distribution of specific pathogens (DS-TB, DR-TB, and NTM) was uniform across sexes and ages (*p* > 0.80).

This suggests that while demographic factors influence susceptibility to active disease, they do not selectively filter the genotypes or resistance profiles of circulating bacilli. The lack of demographic clustering among drug-resistant isolates (DR-TB and Mono-Res INH) reinforces the conclusion that antimicrobial resistance is a systemic challenge driven by prior treatment non-adherence or direct transmission within the community, rather than a biological vulnerability linked to age or sex.

The detection of INH resistance-associated targets (0.89%) in this cohort suggests the potential added value of multiplex molecular screening beyond rifampicin-only assays. However, these findings were not confirmed phenotypically, and resistance may be underestimated if relevant mutations occur outside the inhA and katG targets included in the assay. By utilizing a domestic IVD product with high TKDN, this study supports the mandates of Presidential Regulation No. 67 of 2021 and Presidential Instruction No. 2 of 2022 [12]. The implementation at Prof. Dr. Chairuddin P. Lubis USU Hospital demonstrates that integrating COVID-19-era molecular infrastructure with domestic innovations can accelerate TB case-finding while ensuring health system resilience.

Effective communication between clinical microbiologists and lung specialists remains paramount, particularly when NTM is detected, to differentiate between causative pathogens and transient colonization [13]. The Open PCR system provides the necessary evidence base for these critical clinical decisions, ultimately preventing the misdiagnosis of NTM as MDR-TB and ensuring patients receive targeted, effective therapy.

This study has several limitations. First, no reference standard or comparator assay, such as mycobacterial culture, line probe assay, Xpert MTB/RIF Ultra, phenotypic drug susceptibility testing, or sequencing, was performed; therefore, diagnostic accuracy metrics such as sensitivity, specificity, positive predictive value, negative predictive value, and agreement could not be assessed. Second, detections of INH resistance-associated targets and NTM were based solely on the Open PCR assay and were not independently confirmed, so false-positive or false-negative results cannot be excluded. Third, the assay reports NTM without species-level identification, limiting clinical and epidemiological interpretation. Therefore, the current findings should be interpreted as evidence of molecular NTM detection rather than confirmed NTM disease. Fourth, no clinical, radiological, HIV, or comorbidity data were available, preventing distinction between molecular detection, colonization, and confirmed disease. Fifth, the higher positivity observed in tertiary facilities may reflect referral bias and greater disease severity rather than assay-related differences. Finally, the study period was relatively short and should be interpreted as an implementation snapshot rather than a full epidemiological representation.

## 5. Conclusions

This study demonstrates the feasibility of implementing a domestic multiplex Open PCR system within a decentralized referral network in Medan. The assay enabled simultaneous molecular detection of MTB, INH resistance-associated targets, and NTM-related signals in sputum samples from presumptive pulmonary TB patients. These findings suggest potential operational value for complementing rifampicin-only molecular screening, particularly in referral settings. However, the absence of comparator methods, species-level NTM identification, and clinical correlation limits definitive conclusions regarding diagnostic accuracy and clinical impact. Further studies incorporating culture, sequencing, phenotypic confirmation, and clinical follow-up are needed before broader policy recommendations can be made.

## Figures and Tables

**Table 1:** Demographic characteristics of suspected TB patients.

Demographic Characteristics	*n* (%)
Total cases	1569 (100.00%)
Sex	
Female	899 (57.30%)
Male	670 (42.70%)
Age, years	
Infant (0–2)	9 (0.57%)
Child (3–12)	45 (2.87%)
Adolescent (13–18)	72 (4.59%)
Young Adult (19–24)	129 (8.22%)
Adult (25–44)	365 (23.26%)
Middle Aged (45–64)	633 (40.34%)
Elderly (>65)	316 (20.14%)

**Table 2:** Open PCR system diagnostic outcome.

	Overall *n* = 1569 ^1^	Female *n* = 670 ^1^	Male *n* = 899 ^1^	*p*-Value ^2^
Variable				
Age (years)	50.0 (32.0, 62.0)	49.0 (28.0, 61.0)	51.0 (36.0, 63.0)	0.001
Open PCR Diagnostic Outcome				0.040
Negative	1148 (73.2%)	516.0 (77.0%)	632.0 (70.3%)	
DS-TB	396.0 (25.2%)	143.0 (21.3%)	253.0 (28.1%)	
DR-TB	2.0 (0.1%)	1.0 (0.1%)	1.0 (0.1%)	
Mono Res INH	14.0 (0.9%)	6.0 (0.9%)	8.0 (0.9%)	
NTM	7.0 (0.4%)	3.0 (0.4%)	4.0 (0.4%)	
TB + NTM	2.0 (0.1%)	1.0 (0.1%)	1.0 (0.1%)	

^1^ Median (Q1, Q3); *n* (%). ^2^ Wilcoxon rank sum test: Fisher’s exact test.

## Data Availability

The data presented in this study are available on reasonable request from the corresponding author. The data are not publicly available due to patient privacy and institutional ethical restrictions.

## Supplementary Materials

The following supporting information can be downloaded at: https://www.mdpi.com/article/10.3390/tropicalmed11060168/s1. Data S1: Participating healthcare facilities in Medan, Data S2: Open PCR system diagnostic results stratified by healthcare facility.

## Abbreviations

The following abbreviations are used in this manuscript:

Ct	Cycle Threshold
DNA	Deoxyribonucleic acid
DR-TB	Drug-Resistant Tuberculosis
DS-TB	Drug-Susceptible Tuberculosis
inhA	Isoniazid-A (gene target)
INH	Isoniazid
IVD	In Vitro Diagnostic
JEMM	Joint External Monitoring Mission
katG	Catalase-peroxidase (gene target)
LPA	Line Probe Assay
MDR-TB	Multidrug-Resistant Tuberculosis
MTB	*Mycobacterium tuberculosis*
NGS	Next-Generation Sequencing
NTM	Nontuberculous Mycobacteria
PCR	Polymerase Chain Reaction
RIF	Rifampicin
TB	Tuberculosis
TKDN	Tingkat Komponen Dalam Negeri (Domestic Component Level)
WHO	World Health Organization
