# Supplementary material for: Implementation of Open PCR System for the Detection of TB/DR-TB and NTM in Sputum Samples from Suspected Pulmonary Tuberculosis Patients in Medan, Indonesia
Source: Trop Med Infect Dis. 2026 Jun 18;11(6):168. doi: 10.3390/tropicalmed11060168 (PMC13307877; doi:10.3390/tropicalmed11060168)
Supplement: Supplementary file 1 [file tropicalmed-11-00168-s001.zip › Supplementary Data 2. Facility_Breakdown.pdf]

Supplementary Data 2. Open PCR system diagnostic results based on healthcare facility

**Diagnostic Outcome**	**Overall** N = 1,569[1]	**Clinic / Sub-center** N = 19[1]	**Hospital** N = 830[1]	**Primary Care (Puskesmas)** N = 720[1]
**PCR Result**				
Negative	1,148 (73%)	12 (63%)	552 (67%)	584 (81%)
DS-TB	396 (25%)	7 (37%)	258 (31%)	131 (18%)
Mono Res INH	14 (0.9%)	0 (0%)	11 (1.3%)	3 (0.4%)
NTM	7 (0.4%)	0 (0%)	6 (0.7%)	1 (0.1%)
TB+NTM	2 (0.1%)	0 (0%)	2 (0.2%)	0 (0%)
DR-TB	2 (0.1%)	0 (0%)	1 (0.1%)	1 (0.1%)

[1]n (%)
